# Editorial for the Special Issue “Healthcare-Associated Infections and Antimicrobial Therapy”

**DOI:** 10.3390/microorganisms13040920

**Published:** 2025-04-16

**Authors:** Petros Ioannou, Diamantis P. Kofteridis

**Affiliations:** 1 School of Medicine, University of Crete, PC 71003 Heraklion, Greece; 2 Internal Medicine Department, University Hospital of Heraklion, PC 71110 Heraklion, Greece

Hospital-acquired infections occur frequently among hospitalized patients and are associated with a significant increase in morbidity and mortality [1,2]. The most common hospital-acquired infections include bloodstream, respiratory tract, and urinary tract infections [3]. The epidemiology and microbiology of hospital-acquired infections are changing, mostly due to increasing antimicrobial resistance rates [4]. The problem of increasing antimicrobial resistance leaves few therapeutic options, leading to the revival of old antibiotics or the use of antimicrobial combinations in difficult-to-treat pathogens [5,6]. This Special Issue of *Microorganisms* focuses on recent research on healthcare-associated infections and antimicrobial therapy.

In their article, Taha et al. evaluate the phenotypic and genotypic detection of hypervirulent *Klebsiella pneumoniae* (hvKp) isolated from hospital-acquired infections at Tanta University hospitals [7]. The study involved 300 ICU (intensive care unit) patients. The samples were grown on different agar plates, and standard microbiological techniques were used for phenotypic identification. The hvKp strains were confirmed using the Vitek-2 system, string tests, and tissue culture assays for biofilm formation and polymerase chain reaction (PCR) tests for the identification of capsular genes (K1, K2, and K57) and virulence genes (rmpA, rmpA2, and iuc A). In total, 57 *K. pneumoniae* isolates were isolated, with 21 (36.8%) of them being hvKp and 36 (63.15%) being cKp. The antimicrobial resistance in the cKp group was notably higher. There was a significant difference in biofilm formation between cKp and hvKp isolates. Finally, the string test showed 100% sensitivity and negative predictive value for the detection of hvKp.

Nguyen et al. evaluated the stability of flucloxacillin (FLU) solutions under different storage conditions to assess whether they could be used for outpatient antimicrobial therapy [8]. Their research investigated how temperature and storage duration affected the concentration and pH of FLU solutions. They found that FLU solutions were relatively stable for 24 h at 33 °C. However, longer refrigeration periods followed by exposure to higher temperatures (33 °C and 37 °C) led to a decrease in FLU concentration. Degradation was more pronounced at 37 °C, with a significant drop in concentration and visible changes such as yellowing and precipitate formation. They concluded that FLU could be prepared in saline without buffer and could be administered continuously for 24 h for OPAT if the temperature of the solutions did not exceed 33 °C and if the pH of the FLU solution were higher than 5.9 after reconstitution.

In their article, Saoudi et al. explored methods to improve the effectiveness of antibiotics, particularly against Gram-negative bacteria with multidrug resistance [9]. More specifically, the study focused on the potential of Origanum vulgare essential oil to provide a synergistic or additive effect with antimicrobials, enhancing their ability to fight infections by multidrug-resistant Gram-negative bacteria. To this end, this essential oil led to a reduction of up to 98.4% in bacterial minimum inhibitory concentrations and showed potent activity against biofilms.

Almeida et al. introduced a machine learning (ML)-based approach utilizing molecular descriptors representing the physiochemical attributes of CYP51/ERG11 protein isoforms [10]. Their results show that descriptors related to the composition of amino acids and their combination of hydrophilicity and hydrophobicity can explain the differences between the resistant non-wild-type (NWT) and the wild-type (WT, nonresistant) protein sequences. Thus, this study shows the potential of ML to reveal nuanced patterns in CYP51/ERG11 sequences and provide important molecular signatures that could help in drug development and the computational screening of resistant and nonresistant fungal lineages.

Gatti et al., in their study, investigated the effect of inflammatory burden (assessed by C-reactive protein (CRP), procalcitonin (PCT), and interleukin-6 (IL-6)) on voriconazole (VRC) exposure in 39 pediatric patients requiring allogeneic hematopoietic cell transplantation (HCT) where therapeutic drug monitoring was performed [11]. The study found that increased inflammatory markers were linked to changes in VRC pharmacokinetics, with a higher inflammatory burden leading to drug overexposure. The findings highlight the need for individualized drug dosing and therapeutic drug monitoring. The study also emphasizes the significance of monitoring inflammatory markers as a potential tool in adjusting voriconazole therapy in pediatric HCT patients.

Ocampo et al., in their study, explored the frequency of device-associated infections (DAIs) caused by resistant bacteria, their predictors, and 30-day all-cause mortality in patients with and without COVID-19 [12]. In total, 1521 patients were included, with 1033 having COVID-19. Carbapenem-resistant Enterobacteriaceae (CRE) infections were the most common during the study (*n* = 98; 9.9%), while patients with COVID-19 were more likely to have a higher frequency of metallobeta-lactamase-producing CRE infection (*n* = 15; 33.3%) compared to those without the disease (*n* = 3; 13.0%). A long stay in the ICU, diabetes, and mechanical ventilation were predictors of CRE infection in patients with COVID-19, while the mortality rate was 60.3%. These findings call for heightened vigilance in infection prevention strategies and tailored antimicrobial therapies in these high-risk patients.

In the study by Biros et al., antimicrobial use in a Greek tertiary university hospital during the COVID-19 pandemic is examined [13]. The investigators evaluated the frequency of antimicrobial prescriptions, the factors influencing their use, and how these might have contributed to antimicrobial resistance. Antibiotics were frequently prescribed to COVID-19 patients, even though many did not show signs of bacterial infections. This overuse of antibiotics, particularly broad-spectrum agents, poses a significant risk for the development of antimicrobial resistance. Key factors influencing antimicrobial use included the severity of the disease, the presence of comorbidities, and the clinical condition of the patients. The research stresses the importance of rational antibiotic prescribing and the need for stewardship programs to prevent further resistance. Additionally, the study highlights the urgent need for the continued monitoring and responsible use of antimicrobials, especially during global health crises such as the COVID-19 pandemic.

Karlsson et al. report four cases of hip prosthetic joint infection (PJI) caused by *Cutibacterium avidum* in a 30-month period at a single medical center [14]. This pathogen is traditionally considered a rare cause of PJIs but is gaining recognition as a notable pathogen in such cases. The whole-genome sequencing of the isolates from the four patients revealed that each strain was unique, indicating no common source or outbreak. Antibiotic susceptibility testing showed that all isolates were fully susceptible to the tested antibiotics and lacked known resistance genes. These findings suggest that *C. avidum* may be an underestimated pathogen in PJIs and potentially more prevalent than previously recognized. Enhanced awareness and further research are essential in understanding its role in PJIs and in developing effective prevention and treatment strategies.

Dhayhi et al., in their study conducted in the southwestern province of Saudi Arabia, investigated the bacterial contamination of mobile phones used by healthcare workers (HCWs) in critical care units, including ICUs, pediatric ICUs (PICUs), neonatal ICUs (NICUs), and cardiac care units (CCUs) [15]. The investigators collected 157 samples from HCWs’ mobile phones across a central hospital (CH) and two peripheral hospitals (PHs). The findings revealed that 81.81% of physicians’ and 75.32% of nurses’ mobile phones were contaminated with bacteria. Contamination rates in the CH were 69.56% in ICUs, 80.95% in PICUs, and 70.27% in NICUs. In the PHs, contamination rates were 78.26% in ICUs, 88.88% in NICUs, and 66.66% in CCUs. Overall, mobile phone contamination rates were 72.11% in the CH and 81.13% in the PHs. These results underscore the necessity for the routine disinfection of HCWs’ mobile phones to mitigate the risk of healthcare-associated infections (HAIs). Implementing strict disinfection protocols can significantly reduce the spread of bacterial pathogens in healthcare settings.

Boix-Palop et al., in their study, evaluated the diagnostic performance of plasma Lipocalin-2 (LCN2) concentrations in adult patients with community-acquired pneumonia (CAP) to determine its etiology, severity, and prognosis [16]. This prospective observational study included 130 patients: 71 with bacterial CAP, 42 with CAP of unknown origin, and 17 with viral CAP. Upon admission, plasma LCN2 levels were measured using a modified enzyme immunoassay. The results indicated that LCN2 concentrations were higher in bacterial CAP compared to non-bacterial CAP (122.0 vs. 89.7 ng/mL, *p* = 0.03). However, LCN2 demonstrated limited ability to distinguish between bacterial and non-bacterial CAP. Notably, an LCN2 cutoff of ≥204 ng/mL predicted pneumococcal bacteremia with an AUROC of 0.74 (sensitivity, 70%; specificity, 79.1%). Furthermore, LCN2 levels correlated with severity scores (CURB-65 and PSI), showing a significant linear trend from low-risk to high-risk groups (*p* < 0.001 and 0.001, respectively). These findings suggest that LCN2 has limited utility in differentiating CAP etiology, but it may serve as a valuable biomarker for assessing disease severity in adult patients with CAP.

Schinas et al., in their review, provide a comprehensive overview of diagnostic stewardship (DS), discussing its importance, potential challenges, and future directions [17]. They underline the need for resident physicians to understand DS principles and integrate them into their clinical practice from the very start of their careers. This review also highlights the role of stakeholders in implementing DS. The authors conclude that DS is not just a clinical tool but a philosophy of care that is required for a more responsive, humane, and effective healthcare system.

This Special Issue includes many interesting papers that can increase understanding and aid in decision making among clinicians caring for patients with healthcare-associated infections. We thank all the reviewers for providing valuable feedback during the revisions of the submitted manuscripts and to the *Microorganisms* team for their support during this time with this Special Issue. Lastly, we thank the authors of the published manuscripts for their contributions.
